# P2Y_14_ receptor has a critical role in acute gouty arthritis by regulating pyroptosis of macrophages

**DOI:** 10.1038/s41419-020-2609-7

**Published:** 2020-05-26

**Authors:** Hanwen Li, Wenjiao Jiang, Shumin Ye, Mengze Zhou, Chunxiao Liu, Xiping Yang, Kun Hao, Qinghua Hu

**Affiliations:** 1 State Key Laboratory of Natural Medicines, Key Laboratory of Drug Metabolism and Pharmacokinetics, China Pharmaceutical University, Nanjing, 210009 PR China; 2 Department of Pharmacology, School of Pharmacy, China Pharmaceutical University, Nanjing, 211198 PR China

**Keywords:** Acute inflammation, Acute inflammation

## Abstract

Nod-like receptor protein 3 (NLRP3)-mediated pyroptosis has a causal role in the pathogenesis of gout. P2Y_14_ receptor (P2Y_14_R) distributed in immune cells including macrophages is a Gi-coupled receptor that inhibits the synthesis of cAMP, which has been regarded as a potential regulator of inflammatory response. Nevertheless, the role of P2Y_14_R in MSU-induced pyroptosis of macrophages involved in acute gouty arthritis is still unclear. In our present study, P2Y_14_R knockout (P2Y_14_R-KO) disrupted MSU-induced histopathologic changes in rat synoviums, accompanied with a significant inhibition of pyroptotic cell death characterized by Caspase-1/PI double-positive and blockade of NLRP3 inflammasome activation in synovial tissues, which was consistent with that observed in in vitro studies. Owing to the interaction of NLRP3 inflammasome and cAMP, we then investigated the effect of adenylate cyclase activator (Forskolin) on macrophage pyroptosis and gout flare caused by MSU stimulation. The reversal effect of Forskolin verified the negative regulatory role of cAMP in MSU-induced pyroptosis. More importantly, adenylate cyclase inhibitor (SQ22536) intervention led to a reversal of protection attributed to P2Y_14_R deficiency. Findings in air pouch animal models also verified aforementioned experimental results. Our study first identified the role of P2Y_14_R/cAMP/NLRP3 signaling pathway in acute gouty arthritis, which provides a novel insight into the pathological mechanisms of pyroptosis-related diseases.

## Introduction

As one type of inflammatory arthritis, gout is characterized by intense pain, swollen joints and active inflammation symptom^1,2^. Clinically, gout is commonly considered as a prototypical inflammatory disease caused by excessive serum level of uric acid and deposition of monosodium urate (MSU) crystal in joints^3^. Notably, the formation of MSU crystals would lead to the disorder of purine nucleotide catabolism as well as the activation of NLRP3 inflammasome, followed by the maturation in caspase-1-mediating pyroptosis of macrophages^4^. And in the following pyroptosis process, the breakage of cell membrane causes severe leakage of cell contents and inflammatory cytokines to elicit excessive inflammatory reactions^5^. Preventing pyroptotic cell death has been regarded as an effective therapeutic strategy for treatment of acute gouty arthritis^6^.

As a kind of transmembrane receptor family, purine receptors could selectively bind to extracellular nucleoside or nucleotide to govern widely multiple physiological functions and immune response process^7^. Purine receptors are usually classified as adenosine (P1) and nucleoside (P2) receptors. The latter can be further divided into ligand-gated ion channel receptors (P2X receptors, P2XR) and G-protein-coupled receptors (P2YR)^8^. As a member of G-protein-coupled receptors, P2Y_14_R could be activated by uridine diphosphate glucose (UDP) and uridine diphosphate glucose sugars (UDP-sugars) to initiate subsequent signal transduction pathway via Gi/o coupled protein^9^. It was reported that the activation of P2Y_14_R might be involved in the regulation of immune inflammatory stress^10,11^. Recent studies proposed that MSU could induce overexpression of P2Y_14_R with a significant increase in the release of inflammatory cytokines in human keratinocytes, suggesting the role of P2Y_14_R in the MSU-induced immune inflammatory responses^12^. Notably, a recent study demonstrated that the inducible P2Y_14_R played an important role in LPS and poly (I:C)-induced immune response in Japanese flounder head kidney macrophages^10^. The previous study from our group also showed that the anti-inflammatory activities of novel P2Y_14_R antagonists and further that targeting P2Y_14_R by a series of antagonists partially protects macrophages against MSU-induced inflammatory stimulus^13^. However, how P2Y_14_R might influence inflammatory responses of macrophages remains incompletely defined. As P2Y_14_R induction inhibits adenylyl cyclase to influence production cAMP through Gi, which negatively regulates NLRP3 inflammasome^14^, we investigated the essential role of P2Y_14_R receptor in acute gouty arthritis and explored the possible interaction of P2Y_14_R and MSU-induced pyroptosis centered on cAMP/NLRP3 signals.

## Methods

### Reagents

Uric acid sodium was purchased from Sigma Aldrich (St. Louis, USA). Forskolin and SQ22536 were purchased from MedChemExpress (USA). Phorbol 12-myristate 13-acetate (PMA), propidium iodide (PI), 4’, 6-diamidino-2-phenylindole (DAPI), and Antifade Mounting Medium were provided by Keygen biotech (Nanjing, China). siRNA for transfection was obtained from Genepharma (Shanghai, China). Lipofectamine 2000 was derived from Invitrogen. Caspase-1 Detection Kit was supplied from ImmunoChemistry Technologies (USA). NLRP3 (bs-10021R), ASC (bs-6741R), caspase-1 (bs-10442R), and β-actin (bs-0061R) antibodies for western blot were obtained from Bioss (Beijing, China). NLRP3 (sc-34410) and ASC (sc-22514-R) primary antibodies used for immunofluorescence were obtained from Santa Cruz Biotechnology (USA). Donkey Anti-Goat IgG H&L (Alexa Fluor 488) (ab150129) and Donkey Anti-Rabbit IgG H&L (Alexa Fluor 647) (ab150075) second antibodies used for Immunofluorescence were obtained from Abcam (USA). All cell culture supplement components were obtained from Gibco (Waltham, MA, USA).

### Preparation of MSU crystal

Uric acid dissolved in double-distilled water containing NaOH (1 m) was then adjusted to pH 7.2 and stored overnight at 4˚C for crystal formation. Subsequently, the precipitate was filtered from the solution and dried at 70˚C for 4 h. After sifted with a 200 mesh metal screen, a fine powder was sterilized by heating at 180˚C for 2 h and stored in sterile conditions. Prior to administration, the MSU crystal were resuspended in sterile PBS at 20 mg/ml.

### Animal

All animal studies were approved by the Animal Ethics Committee of China Pharmaceutical University and carried out in accordance with the Guide for the Care and Use of Laboratory Animals. Male adult Sprague–Dawley rats (6 weeks, 180–220 g) were purchased from Qinglongshan Experimental Animal Center (Nanjing, License number: SCXK (Su) 2017-0001). Rats were maintained in a room at 22–24 °C under a humidity of 55 ± 5% and a standardized light–dark cycle. P2Y_14_R-knockout Sprague–Dawley rats (6 weeks, 180–220 g) provided by BIOCYTOGEN (Beijing, China) were subjected to genotype identification prior to experiments. The related data were exhibited in the supplemental Fig. 1. Animals were given standard chow ad libitum and allowed to acclimatize standard conditions of the animal center a week prior to experiments.

Animals were randomly assigned to each group in this study (*n* = 6). The acute gouty arthritis model was established by injection into ankle joint cavity with 100 μl MSU crystals (500 μg/ml) dissolved in sterile PBS. Intra-articular administration of Forskolin and SQ22536 (10 mg/kg) were given to animals three times at 0, 24, 48 h prior to MSU induction. In order to avoid irritation caused by repeated injections, last treatments of Forskolin or SQ22536 were given to animals at the same time with modeling liquid. In contrast, the control group was injected with the same volume of sterile PBS into ankle joint cavity. The injected ankle perimeter of rats with string was determined at 0, 2, 4, 8, 12, 24 h after MSU stimulation. Animal processing and data analysis were performed blindly. Each measurement was duplicated reproducibly.

### Cell transfection

THP-1 cells derived from American Type Culture Collection (Manassas, VA, USA) were cultured at 37˚C under 5% CO_2_ in RPMI 1640 supplemented with 10% fetal bovine serum (Gibco, USA), 100 IU/ml penicillin, and 100 IU/ml streptomycin. Transfection was performed followed by pre-differentiation for 48 h in supplemented RPMI 1640 containing 100 ng/ml PMA. P2Y_14_R siRNA (P2Y_14_-homo-1129: 5’–3’: CCUUAAGUCHHAAUTT. 3’–5’: AUUCCGACUUGACUUAAGGTT) was used for transient transfection in the presence of lipofectamine 2000. The transfected cells were cultured for 48 h prior to the following treatments. Forskolin and SQ22536 were respectively dissolved in DMSO at the concentration of 10 mm as stock solutions. In all, 15 min pre-treatments of Forskolin and SQ22536 were exposed to THP-1 cells followed by MSU model (500 µg/ml) for 12 h. Each measurement was duplicated reproducibly.

### Caspase-1/PI double staining

For pyroptosis analysis, active Caspase-1 and PI fluorescence of samples were measured using flow cytometry. Active caspase-1 was detected with FLICA 660 Caspase-1 Detection Kit (ImmunoChemistry Technologies, USA) and PI staining was used to assess the integrity of cellular membrane. In brief, for THP-1 samples, the cell density was adjusted to 2–5 × 10^5^/ml according to the manufacturer’s instructions. The FLICA 660 working solution was added into cell suspension at a ratio of 1:30-1:60 (v/v). The cells were incubated at 37˚C for 45 min. After incubation, the cells were washed with wash Buffer and were centrifuged at 12,000 rpm for 5 min at room temperature. Next, the cell supernatants were discarded and the cells were resuspended in wash Buffer and gently mixed. Then the PI was added into cell suspension 5 min prior to analyzing using flow cytometry.

For synovial samples, synovial tissue was extracted and washed with PBS 24 h after MSU challenge. Tissues were cut into pieces and incubated with 0.2% Type III collagenase for 2 h at 37˚C followed by 0.25% Trypsin for 0.5 h at 37˚C. After digestion, the liquid was centrifuged at 1000 rpm for 10 min and was filtered to collect the cells. Following operations of synovial samples were consistent with the above description.

### Immunofluorescence

After MSU stimulation, the cells were 4% paraformaldehyde fixed for 20–30 min. Permeabilization was performed with 0.3–0.5% Triton X-100 for 20–30 min. When blocking for 1 h to avoid non-specific protein interactions, the samples were incubated with the primary antibody at a 1:50 dilution for immunofluorescence staining overnight at 4˚C. The secondary antibodies were Donkey Anti-Goat IgG H&L (Alexa Fluor 488) and Donkey Anti-Rabbit IgG H&L (Alexa Fluor 647) used at a 1/200 dilution for 1.5 h. Fluorescent images were visualized by confocal laser scanning microscope (Fluoview, FV1000, Olympus, Japan).

### Biochemical assay

The detection of intracellular cAMP was performed using cAMP-Glo Assay (Promega, USA). The levels of IL-1β in culture supernatants were measured using human IL-1β ELISA kit (NeoBioScience, China). All procedure were conducted strictly according to the manufacturer’s instructions and analyzed on a grating microplate reader (Corona SH-1000Lab, Hitachi, Ltd, Japan).

### Histopathological examination

The synovium tissue samples were collected immediately, fixed in the 4% paraformaldehyde solution and then embedded with paraffin. In total, 5 μm paraffin sections was dewaxed in xylene, rehydrated through ethanol and stained with hematoxylin and eosin (H&E) according to the standard protocol. Then the histopathological evaluation was performed.

### Western blot

The synovium tissue and THP-1 cells were collected and lysed in a RIPA buffer (Beyotime, China). The total protein concentration was measured using a BCA protein assay kit (Beyotime, China). Samples containing ~50 mg protein was separated by 8–12% sodium dodecyl sulfate-polyacrylamide gel electrophoresis (SDS-PAGE) followed by the transference to polyvinylidene fluoride (PVDF) membranes (Millipore Corporation, MA, USA). Subsequently, PVDF membranes were blocked with 5% (w/v) non-fat milk in TBST buffer for 2 h at room temperature and treated with corresponding primary antibodies (1:500 to 1:1000) overnight at 4˚C. The membranes were washed three times with Tris buffer saline-Tween20 (TBST), followed by incubation with appropriate horseradish peroxidase-conjugated secondary antibodies (1:1000 to 1:2000) for 2 h. Finally, protein bands were visualized with an enhanced chemiluminescence (ECL) system (Keygen Biotech, China) and scanned with a Chemiluminescence imaging system (Gel Catcher 2850, China). The relative optical densities of bands were analyzed with a ChemiScope analysis program.

### Statistical analysis

Data were presented as the mean ± standard deviation (SD). One-way analysis of variance (ANOVA) with Tukey multiple comparison test was performed to compare among the different groups after the assessment of normal distribution and homogeneity of variance test. A *P* < 0.05 was considered to be statistically significant.

## Results

### P2Y_14_R deficiency increased resistance to MSU-induced acute gouty arthritis

**Fig. 1:**
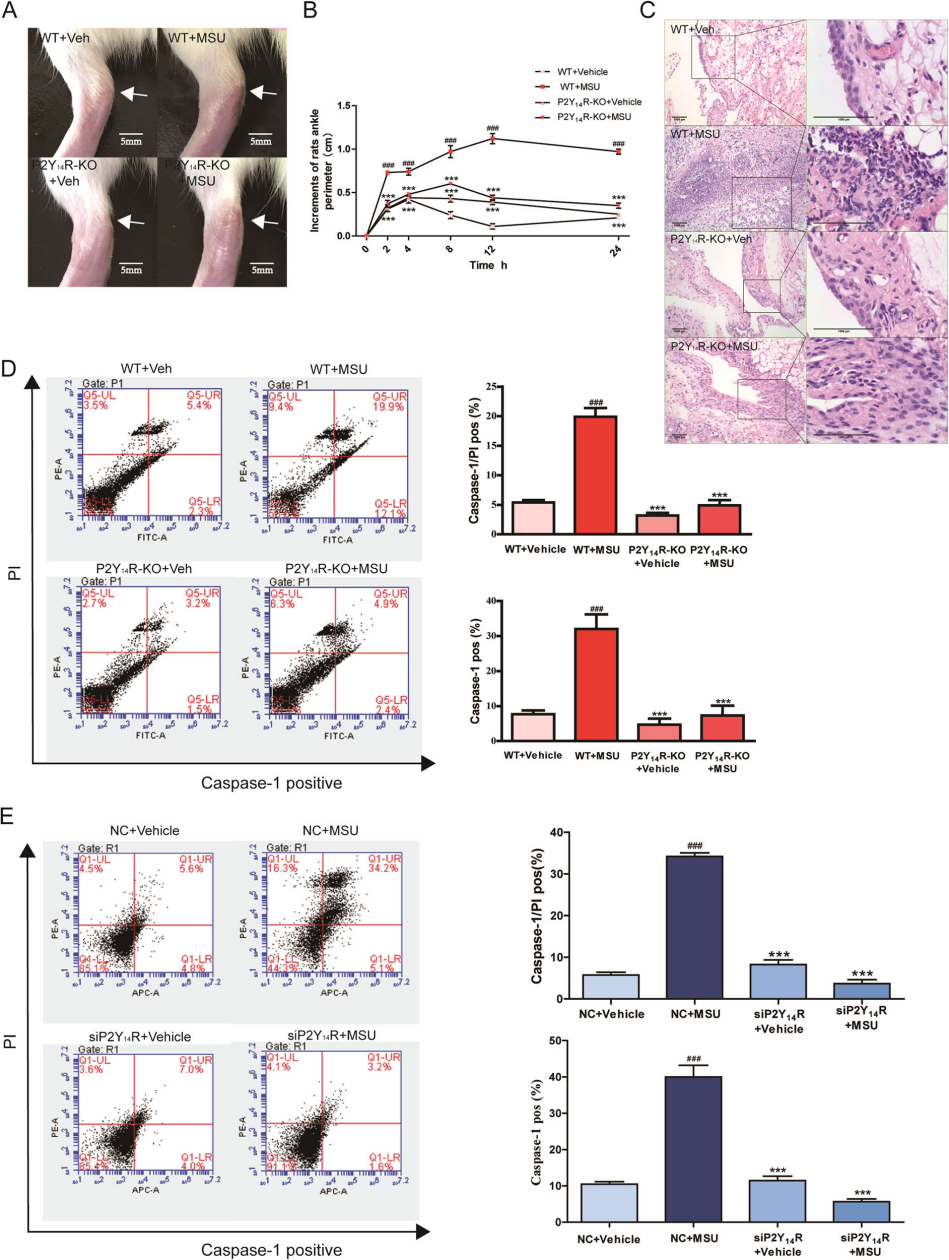
P2Y_14_R could regulate MSU-induced pyroptosis in vivo and in vitro. **a** The acute gouty arthritis was induced by intra-articular injection with MSU solution. P2Y_14_R-KO rats exhibited improved joints swelling caused by MSU. **b** The circumference of rat ankle joint was increased after the injection of MSU in WT rats but not P2Y_14_R-KO rats. The injected ankle joint circumference of each rat was determined at 0, 2, 4, 8, 12, 24 h after MSU stimulation (*n* = 6). **c** Histopathologic evaluation exhibited a remarkable elevation in the inflammatory infiltration of synovial tissue in WT but not P2Y_14_R-KO rats after MSU exposure. **d** The macrophages derived from P2Y_14_R-KO synovium exhibited a lower positivity of active Caspase-1 and PI double staining in pyroptosis assay (*n* = 4). **e** After transfection for 48 h, the THP-1 cells were stimulated by MSU. The rate of pyroptotic cell death was examined with active PI and Caspase-1 double staining by flow cytometry (*n* = 4). P2Y_14_R knockdown led to a decreased rate of pyroptotic cell death. The data were presented as means ± SDs. One-way analysis of variance (ANOVA) with Tukey multiple comparison test was performed. Compared with WT/NC + vehicle group: ^#^*P* < 0.05, ^##^*P* < 0.01, ^###^*P* < 0.001. Compared with WT/NC + MSU group: ^*^P < 0.05, ^**^P < 0.01, ^***^P < 0.001.

First of all, the MSU stimulation was given to wild-type (WT) rats and P2Y_14_R knockout (P2Y_14_R-KO) rats to assess if P2Y_14_R was involved in the provoked inflammatory actions associated with acute gouty arthritis. Intense inflammatory response could result in the ankle joint swelling and an marked inflammatory infiltration^15^. Here, as shown in Fig. 1a, b, P2Y_14_R-KO rats with MSU treatment showed a continuous stability of ankle perimeter during experimental period. In contrast, an obvious elevation in ankle perimeter could be observed in WT rats receiving MSU, suggesting the occurrence of exaggerated joint swelling. In addition, there was also a remarkable difference in the inflammatory infiltration of synovial tissue between WT and P2Y_14_R-KO rats after MSU exposure (Fig. 1c). When compared WT + MSU animals, P2Y_14_R-KO + MSU members progressed to less synovial lesion in histopathological test, as evidenced by significantly suppressed synovial hyperplasia and decreased infiltration of inflammatory cells in synovium.

Given the fact that MSU-induced gouty arthritis pathogenesis was frequently accompanied by pyroptotic onset, the pyroptosis assay was performed to explore the gouty arthritis resistance of P2Y_14_R-KO. PI staining was used for pyroptosis assay, owing to its property of identifying the pyroptosis-induced permeability alteration in cellular membrane. Double staining detection of active Caspase-1 and PI has been performed to evaluate pyroptosis rate^16,17^. Flow cytometry data revealed a significant upregulation occurs in the ratio of PI and Caspase-1 double-positive macrophages obtained from MSU-stimulated model synovium in contrast with those of normal group. However, this positive correlation between MSU exposure and pyroptosis stress was turned out to invalidate in the P2Y_14_R-KO rats treated with MSU (Fig. 1d). Next, we detected the effect of P2Y_14_R knockdown on MSU stimulation in THP-1 cells. We set up a P2Y_14_R knockdown group with the efficient siRNA and lipofectamine 2000. siRNA was successfully transfected into THP-1 cells and then the expression of P2Y_14_R was assessed (Supplementary Figure 2D). Consistent with data in vivo, MSU-induced THP-1 cells exhibited an inhibited pyroptotic rate under P2Y_14_R knockdown with siRNA in pyroptosis assay, consistent with the data in vivo (Fig. 1e). Hence, we proposed that P2Y_14_R might be involved in the pyroptosis death of MSU-induced acute gouty arthritis.

### P2Y_14_R deficiency regulated the NLRP3 inflammasome activation in vivo and in vitro

**Fig. 2:**
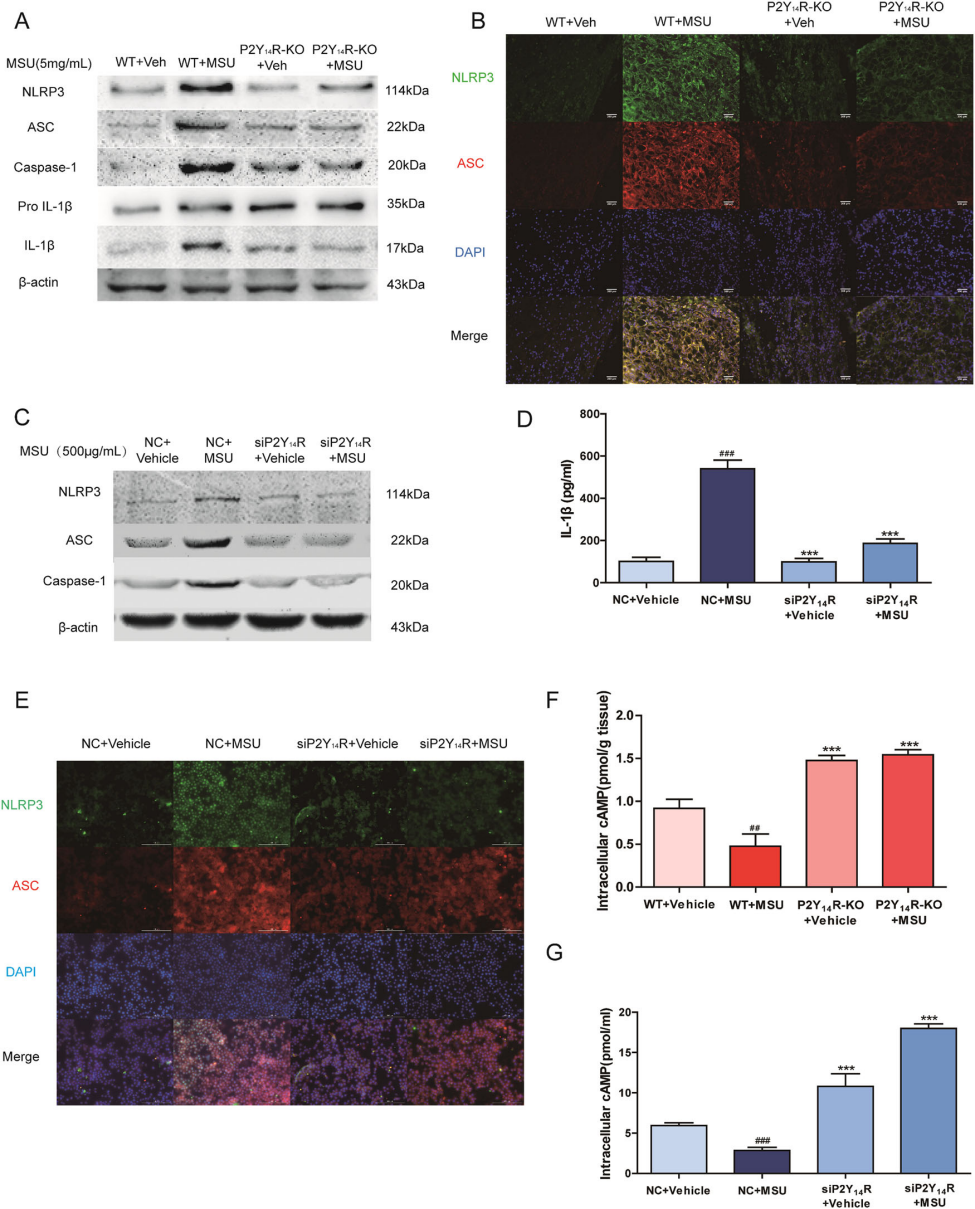
NLRP3 inflammasome activation was involved in P2Y_14_R deficiency. **a** The NLRP3 inflammasome activation and IL-1β mutation in synovium was inhibited in P2Y_14_R-KO rats detected by western blotting. The relative optical density was exhibited in the supplementary materials (*n* = 4). **b** Immunofluorescence assay confirmed that MSU-induced NLRP3 inflammasome activation in synovial tissue of WT but not P2Y_14_R-KO rats. NLRP3 protein was marked with Alexa Fluor 488 (Green). ASC protein was marked with Alexa Fluor 647 (Red). DAPI (Blue) was used to mark the nucleus. **c** The expression of NLRP3 inflammasome activation was inhibited under P2Y_14_R knockdown. P2Y_14_R siRNA was used to transfect THP-1 cells for 48 h, followed by MSU stimulation for 12 h. The relative optical density was exhibited in the supplementary materials (*n* = 4). **d** ELISA kit data showed that the release of IL-1β decreased when P2Y_14_R was knockdown with siRNA. **e** Immunofluorescence assay revealed that MSU administration could not induce the NLRP3 inflammasome activation anymore in siP2Y_14_R THP-1 cells. NLRP3 protein was marked with Alexa Fluor 488 (Green). ASC protein was marked with Alexa Fluor 647 (Red). DAPI (Blue) was used to mark the nucleus. **f** The intracellular cAMP level in synovial tissue increased in P2Y_14_R-KO rats compared WT ones (*n* = 6). **g** The intracellular cAMP level in THP-1 cells increased under P2Y_14_R knockdown (*n* = 4). The data were presented as means ± SDs. One-way analysis of variance (ANOVA) with Tukey multiple comparison test was performed. Compared with WT/NC + vehicle group: ^#^*P* < 0.05, ^##^*P* < 0.01, ^###^*P* < 0.001. Compared with WT/NC + MSU group: ^*^P < 0.05, ^**^P < 0.01, ^***^P < 0.001 (*n* = 4).

Emerging studies reported that a positive correlation existed between NLRP3 inflammasome activation and pyroptosis onset^18^. The activation of NLRP3 inflammasome pathway were evaluated to further elucidate whether NRLP3 inflammasome was involved in the P2Y_14_R-KO resistance to MSU-challenged pyroptosis. Western blot of synovial tissue confirmed that MSU administration induced the NLRP3 inflammasome activation, as increased expressions of NLRP3, ASC, active Caspase-1 and downstream active IL-1β. Although, when compared with WT rats, the facilitative effect of MSU on upregulating NLRP3 signaling disappeared in P2Y_14_R-KO rats (Fig. 2a). This significant difference was also observed in the immunofluorescence assay (Fig. 2b).

Consistently, as shown in the Fig. 2c, the expression of NLRP3, ASC, and active Caspase-1 (p20) were obviously upregulated, implying the complete assembly of NLRP3 inflammasome when exposed to MSU stimulation for 12 h. However, when P2Y_14_R was silenced, the MSU induction did not trigger the NLRP3 signaling anymore in contrast with native control cells, which could be also verified by colocalization of NLRP3 and ASC with confocal laser scanning microscope (Fig. 2e). Immunofluorescence data showed that MSU crystals made higher intensities of NLRP3 and ASC in the NC + vehicle group rather than siP2Y_14_R + vehicle group, implicating that blocking P2Y_14_R expression might prevented the Caspase-1-mediated pyroptosis progression via regulating NLRP3 inflammasome signaling.

### cAMP played a key role in the regulation of NLRP3-mediated pyroptosis

Cyclic adenosine monophosphate (cAMP) has been reported to directly bind with intracellular NLRP3 to promote its ubiquitination and degradation in macrophages^19^. Subsequently, we assayed the synovium levels of intracellular cAMP in WT and P2Y_14_R-KO rats. As shown in the Fig. 2f, P2Y_14_R deficiency resulted in a significant elevation in the intracellular cAMP content in synovial tissue when compared with WT rats. And Fig. 2g demonstrated that the siP2Y_14_R + vehicle THP-1 cell also had a higher intracellular level of cAMP than NC + vehicle ones, suggesting that cAMP metabolism might be regulated by P2Y_14_R.

**Fig. 3:**
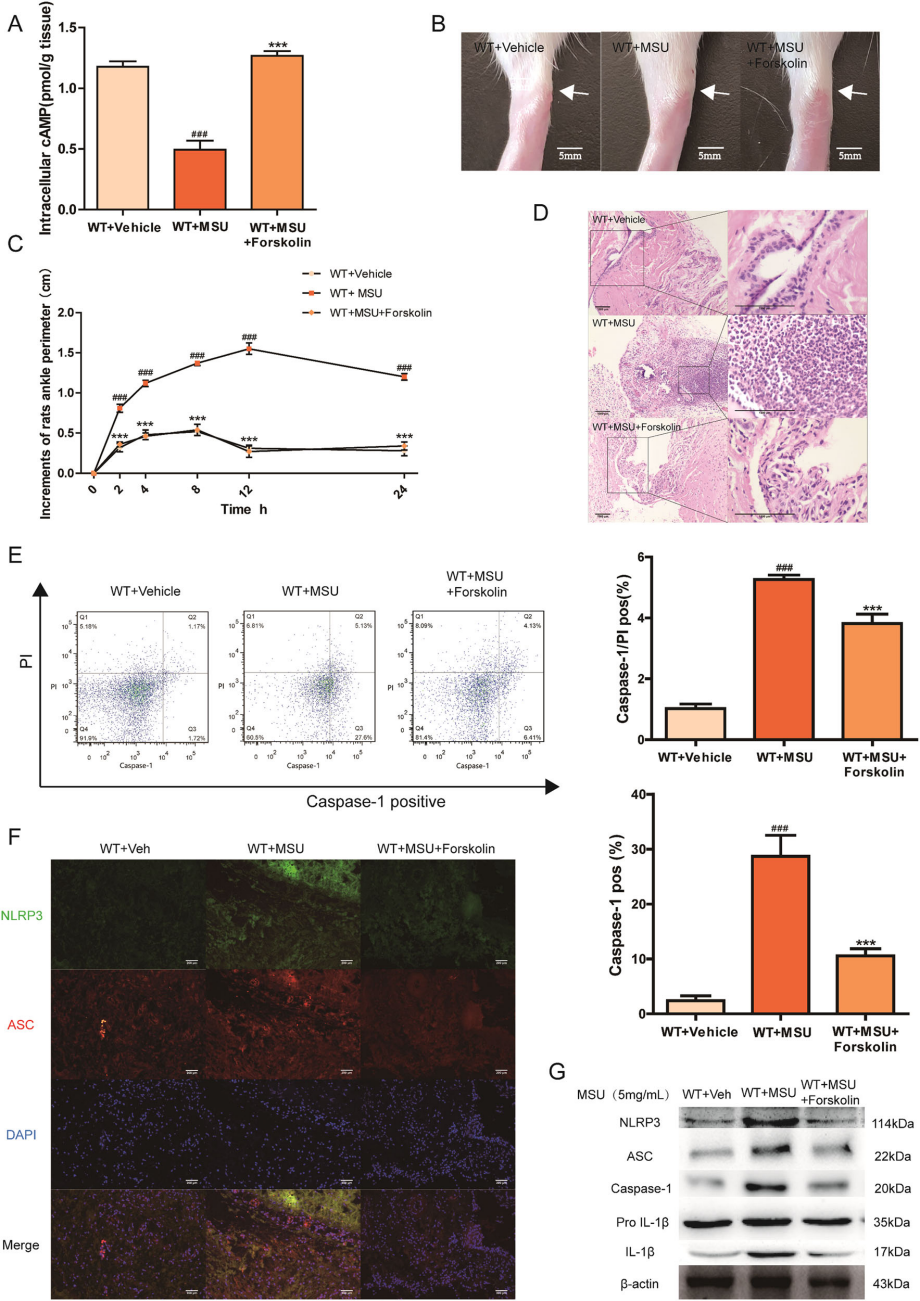
Upregulated cAMP suppressed acute gouty arthritis in vivo. As a potent adenylate cyclase (AC) activator, Forskolin is used to increase the intracellular level of the second messenger cAMP. Intra-articular administration of Forskolin was given to WT rats prior to MSU induction. **a** The intracellular cAMP level in synovial tissue increased significantly after Forskolin treatment (*n* = 6). **b** cAMP elevation induced by Forskolin prevented the joint swelling. Representative photographs to show the swelling of joints are presented. **c** Forskolin-induced cAMP elevation alleviated the injected ankle joint circumference under MSU challenge (*n* = 6). **d** Forskolin treatment efficiently inhibited inflammatory cell infiltration when compared MSU group in histopathologic test. **e** The macrophages derived from synovium of Forskolin-treated rats exhibited a lower positivity of active Caspase-1 and PI double staining in pyroptosis assay by flow cytometry (*n* = 4). **f** Forskolin treatment inhibited the colocalization intensity of NLRP3 and ASC in MSU-stimulated WT animals in immunofluorescence staining. NLRP3 protein was marked with Alexa Fluor 488 (Green). ASC protein was marked with Alexa Fluor 647 (Red). DAPI (Blue) was used to mark the nucleus. **g** Western blotting showed Forskolin treatment inhibited MSU-induced NLRP3 inflammasome activation in WT rats. The relative optical density was exhibited in the supplementary materials (*n* = 4). The data were presented as means ± SDs. Compared with WT + vehicle group: ^#^*P* < 0.05, ^##^*P* < 0.01, ^###^*P* < 0.001. Compared with WT + MSU group: ^*^P < 0.05, ^**^P < 0.01, ^***^P < 0.001.

**Fig. 4:**
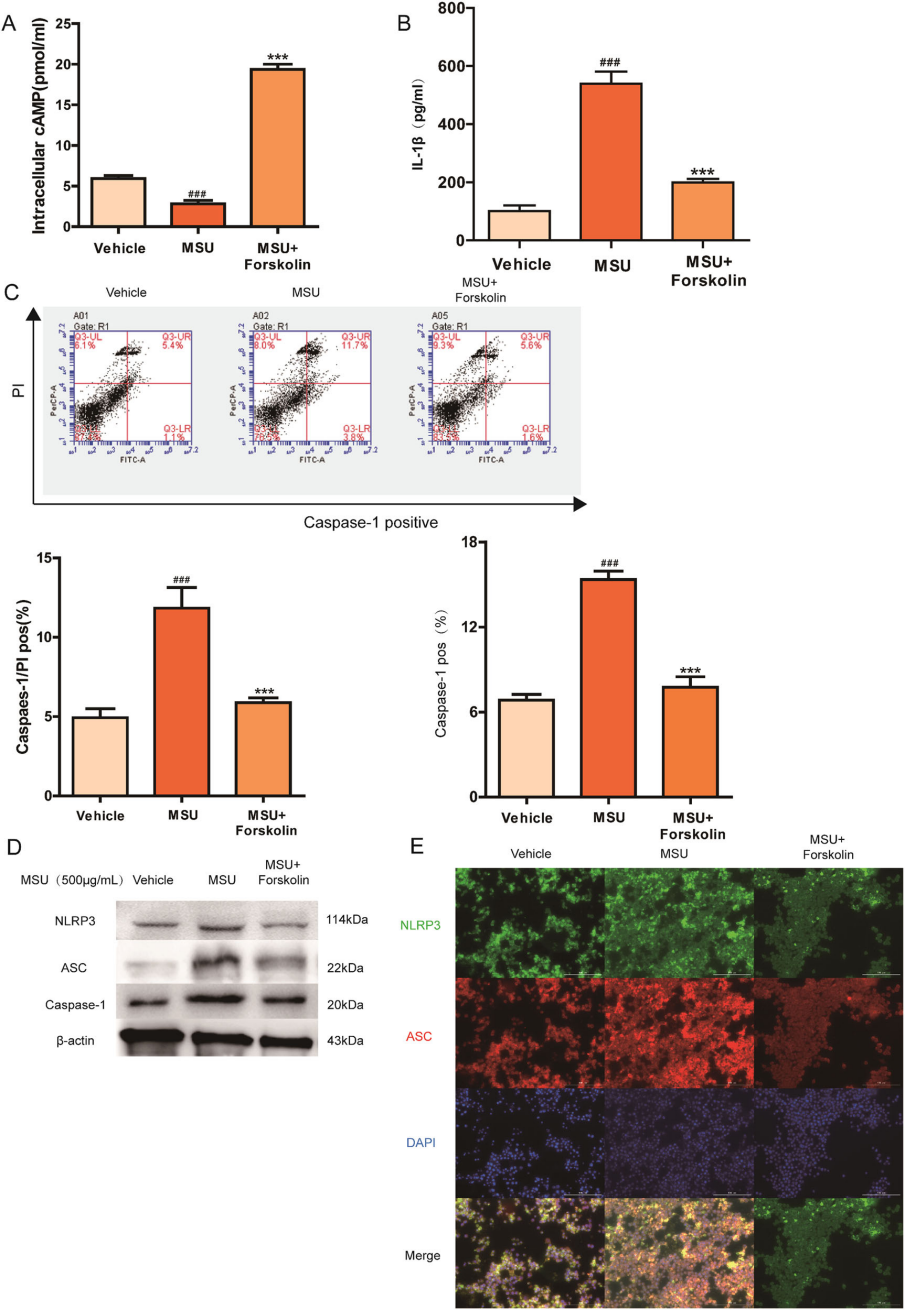
Pyroptosis stress was invalidated once cAMP increased by Forskolin in vitro. 15 min pre-treatment of Forskolin was exposed to THP-1 cells followed by MSU model for 12 h. **a** The intracellular cAMP level in THP-1 cells was verified to rise after Forskolin treatment by cAMP assay kit (*n* = 4). **b** Forskolin-induced cAMP elevation suppressed the release of IL-1β in THP-1 cells (*n* = 4). **c** The THP-1 cells treated with Forskolin presented a decreased rate of pyroptotic cell death in active Caspase-1 and PI double staining by flow cytometry (*n* = 4). **d** Western blotting showed that the enhanced cAMP level attenuated the MSU-induced activation of the NLRP3 signaling pathway in THP-1 cells treated with Forskolin. The relative optical density was exhibited in the supplementary materials (*n* = 4). **e** Immunofluorescence assay confirmed that enhanced cAMP level attenuated MSU-induced NLRP3 inflammasome activation, as evidenced by decreased expression and colocalization of NLRP3 and ASC in THP-1 cells treated with Forskolin. NLRP3 protein was marked with Alexa Fluor 488 (Green). ASC protein was marked with Alexa Fluor 647 (Red). DAPI (Blue) was used to mark the nucleus. The data were presented as means ± SDs. One-way analysis of variance (ANOVA) with Tukey multiple comparison test was performed. Compared with vehicle group: ^#^*P* < 0.05, ^##^*P* < 0.01, ^###^*P* < 0.001. Compared with MSU group: ^*^P < 0.05, ^**^P < 0.01, ^***^P < 0.001.

To further confirm the involvement of cAMP in acute gouty arthritis, we explored the role of cAMP on acute gouty arthritis. As a potent adenylate cyclase (AC) activator, Forskolin is frequently used to raise the intracellular level of cAMP. In MSU-induced animal model, Forskolin treatment efficiently improved the intracellular level of cAMP in synovial tissue when compared with WT + MSU group and significantly prevented the joint swelling and neutrophilic infiltration (Fig. 3a–d). The beneficial effect of cAMP elevation on MSU model was also observed in inhibited pyroptosis rate, as shown by a significant alleviation in the ratio of PI and Caspase-1 double-positive macrophages obtained from synovium (Fig. 3e). In addition, treatment of Forskolin abolished MSU-induced NLRP3 inflammasome activation in WT rats, and inhibited the colocalization intensity of NLRP3 and ASC in MSU-stimulated WT animals (Fig. 3f, g). In THP-1 cells, cAMP upregulation caused by Forskolin could defect against MSU stimulation with lower pyroptotic stress and less IL-1β secretion than THP-1 cells treated with MSU only (Fig. 4h–j). Meanwhile, western blot and immunofluorescence data demonstrated that the enhanced cAMP level attenuated the MSU-induced activation of the NLRP3 signaling pathway, suggesting the crucial role of cAMP in the regulation of P2Y_14_R-mediated gouty arthritis (Fig. 4k, l).

### Decreased cAMP reversed the protective effect of P2Y_14_R deficiency in vivo and in vitro

**Fig. 5:**
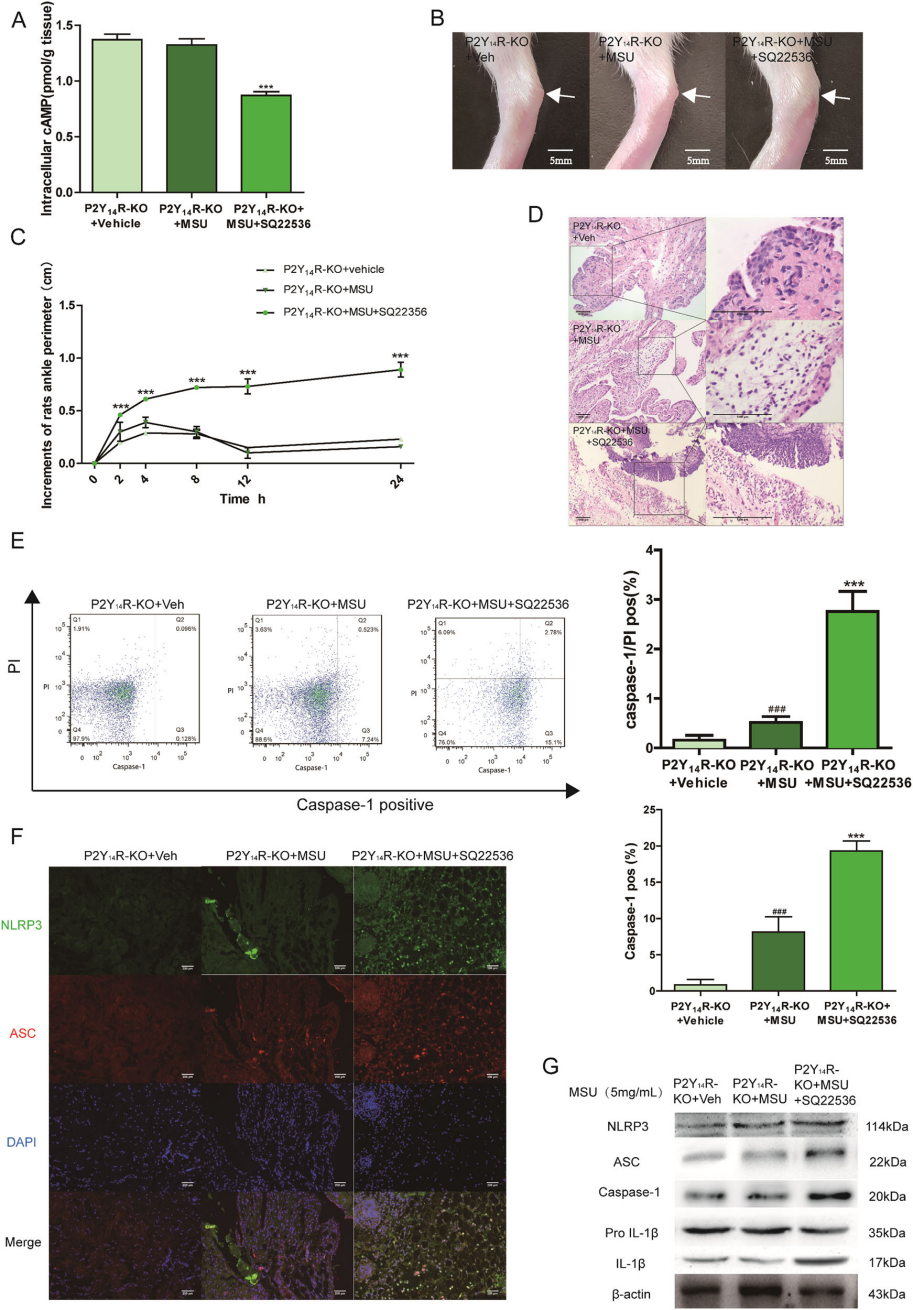
Decreased cAMP exaggerated acute gouty arthritis in P2Y_14_R-KO rats. SQ22536, an adenylate cyclase (AC) inhibitor, was used in our study to reduce cAMP levels in P2Y_14_R-KO rats. Intra-articular administration of SQ22536 was given to P2Y_14_R-KO rats prior to MSU model. **a** The intracellular cAMP level decreased significantly in P2Y_14_R-KO synovial tissue after SQ22536 treatment by cAMP assay kit (*n* = 6). **b** cAMP reduction caused by SQ22536 abolished the effective effect of P2Y_14_R-KO on the joint swelling. Representative photographs to show the swelling of joints are presented. **c** SQ22536-induced cAMP reduction apparently aggravated the injected ankle joint circumference under MSU challenge in P2Y_14_R-KO rats (*n* = 6). **d** SQ22536 treatment exhibited a significant exacerbation of inflammatory cell infiltration in histopathologic evaluation of P2Y_14_R-KO rat synovial tissues. **e** The increment of pyroptosis positivity could be observed in the macrophages derived from P2Y_14_R-KO rat synovium after SQ22536 stimulation by flow cytometry (*n* = 4). **f** SQ22536 treatment enhanced the colocalization intensity of synovial NLRP3 and ASC in MSU-stimulated P2Y_14_R-KO rats in immunofluorescence staining. NLRP3 protein was marked with Alexa Fluor 488 (Green). ASC protein was marked with Alexa Fluor 647 (Red). DAPI (Blue) was used to mark the nucleus. **g** Western blotting showed that the activation of synovial NLRP3 inflammasome signaling was markedly provoked by decreased cAMP in SQ22536-treated P2Y_14_R-KO rats. The relative optical density was exhibited in the supplementary materials (*n* = 4). The data were presented as means ± SDs. One-way analysis of variance (ANOVA) with Tukey multiple comparison test was performed. Compared with P2Y_14_R-KO + vehicle group: ^#^*P* < 0.05, ^##^*P* < 0.01, ^###^*P* < 0.001. Compared with P2Y_14_R-KO + MSU group: ^*^P < 0.05, ^**^P < 0.01, ^***^P < 0.001.

SQ22536, an adenylate cyclase (AC) inhibitor, was used in our study to reduce cAMP levels to investigate the role of intracellular cAMP in the P2Y_14_R-KO protection. Intriguingly, it could be observed that SQ22536 treatment exhibited a significant exacerbation of ankle swelling and neutrophil infiltration with a decreased level of cAMP in synovial tissue (Fig. 5a–d). And the SQ22536 administration was found in Fig. 5e to disrupt the protective effect of P2Y_14_R-KO against MSU stress with the increment of pyroptosis positivity. Consistent with exacerbated pyroptosis, western blot showed that the activation of NLRP3 inflammasome signaling was markedly provoked by decreased cAMP, which was also supported by colocalization immunofluorescence data (Fig. 5f, g).

**Fig. 6:**
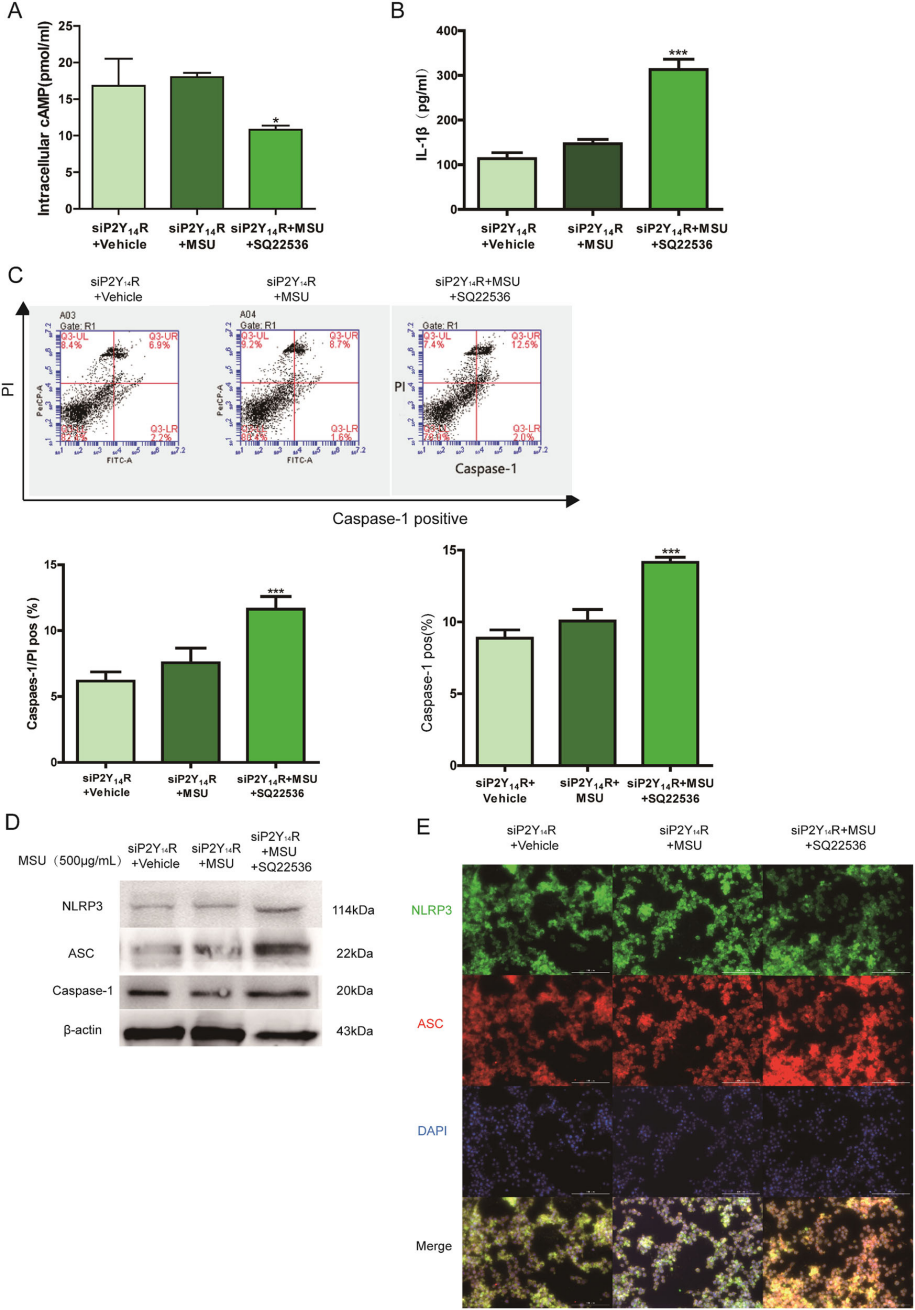
Decreased cAMP reversed the protective effect of P2Y_14_R knockdown in THP-1 cells. P2Y_14_R siRNA was used to transfect THP-1 cells with Lipofectamine 2000 for 48 h. 15 min pre-treatment of SQ22536 was exposed to THP-1 cells followed by MSU model for 12 h. **a** The intracellular cAMP level in THP-1 cells was verified to reduce by cAMP assay kit in siP2Y_14_R THP-1 cells after SQ22536 treatment (*n* = 4). **b** SQ22536-induced cAMP reduction promoted the release of IL-1β in siP2Y_14_R THP-1 cells (*n* = 4). **c** Decreased cAMP in THP-1 cells restored the siP2Y_14_R-derived protective effect on MSU-stimulated pyroptosis with a rising rate of active Caspase-1 and PI double positivity by flow cytometry (*n* = 4). **d** Western blotting showed that the decreased cAMP level promoted the MSU-induced activation of the NLRP3 signaling pathway in THP-1 cells treated with SQ22536 under P2Y_14_R knockdown. The relative optical density was exhibited in the supplementary materials (*n* = 4). **e** Immunofluorescence assay confirmed that decreased cAMP recovered NLRP3 inflammasome activation in THP-1 treated with P2Y_14_R knockdown, as evidenced by increased expression and colocalization of NLRP3 and ASC after SQ22536 administration. NLRP3 protein was marked with Alexa Fluor 488 (Green). ASC protein was marked with Alexa Fluor 647 (Red). DAPI (Blue) was used to mark the nucleus. The data were presented as means ± SDs. One-way analysis of variance (ANOVA) with Tukey multiple comparison test was performed. Compared with siP2Y_14_R + vehicle group: ^#^*P* < 0.05, ^##^*P* < 0.01, ^###^*P* < 0.001. Compared with siP2Y_14_R + MSU group: ^*^P < 0.05, ^**^P < 0.01, ^***^P < 0.001.

Next, we performed corresponding analysis in vitro to further confirm the of involvement of cAMP regulation in P2Y_14_R resistance. As revealed in Fig. 6a, after the treatment with siP2Y_14_R, MSU could not shift the intracellular level of cAMP anymore when compared with siP2Y_14_R + vehicle group. However, after SQ22536 treatment, a decreased cAMP content in THP-1 cells appeared and restored the siP2Y_14_R-derived protective effect on MSU-stimulated pyroptosis, which was confirmed by a notable upregulation in active Caspase-1 positivity as well as IL-1β release (Fig. 6b, c). And western blot and immunofluorescence data in Fig. 6d, e showed that decreased cAMP induced by SQ22536 recovered NLRP3 inflammasome activation under P2Y_14_R knockdown. Hence, we found the intracellular cAMP might be involved in the regulation of P2Y_14_R on NLRP3 inflammasome-mediated acute gouty arthritis.

## Discussion

Gout pathogenesis is accompanied with an elevated urate concentration in serum, which eventually lead to the formation of MSU crystals^4^. Despite the chronic and spontaneous development of MSU deposition in joints of patients with gouty arthritis, direct MSU exposure to rat animal model has been applied in recent studies to establish a feasible model of acute gouty arthritis^15,20,21^. Notably, it has been reported that MSU stimulation can induce NLRP3 inflammasome activation in macrophages with an onset of programmed cell death known as pyroptosis^18,22^. In this study, we revealed a role of P2Y_14_R in regulating pyroptosis process in vitro and in vivo. Specifically, an adenylate cyclase (AC) was found to be involved in the effect of P2Y_14_R on caspase-1-mediated pyroptosis. These findings might attribute to elucidate the metabolic mechanisms of the MSU-induced gouty arthritis.

Nucleotides released from cells as extracellular signaling molecules, could activate cell surface purinergic receptors to trigger a series of physiological processes^23,24^. Among multiple purinergic receptors, P2Y_14_R was acknowledged as a G-protein-coupled receptors (GPCRs) combining with extracellular nucleoside^25,26^. Particularly, unlike other P2Y receptors, P2Y_14_R was merely activated by UDP as well as UDP-sugars, with a potency order of UDP-glucose > UDP-galactose > UDP-glucuronic acid > UDP-*N*-acetylglucosamine^11,27^. And UDP-glucose acted as a damage-associated molecular pattern molecule (DAMP), which mainly released by injured sites of body, suggesting the relationship between P2Y_14_R induction and inflammatory reaction^9^. For example, patients with fibrosis and asthma were reported to exhibit elevated UDP-glucose levels in their lungs^28,29^. And P2Y_14_R activation by UDP-glucose in airway epithelial cells led to intracellular Ca^2+^ concentration and IL-8 secretion^30^. Furthermore, it was proposed that UDP-glucose was able to stimulate IL-8 production and enhanced neutrophil chemotaxis via the presence of P2Y_14_R in a human endometrial epithelial cell line^31^. Hence, among DAMPs, UDP-glucose has recently emerged as a potential extracellular signaling molecule^9,32^.

Meanwhile, there seemed to be an association between P2Y_14_R overexpression and inflammation exaggeration. P2Y_14_R mRNA expression is upregulated by lipopolysaccharides (LPS), implying its role in mediating inflammation^33^. Besides, the mRNA and protein expression of P2Y_14_R in the brain microvascular endothelial cells (BMECs) were also upregulated in oxygen-glucose-deprivation (OGD)-induced injury^34^. And it was reported that MSU crystals induced an increased expression of P2Y_14_R in normal human epidermal keratinocytes in a dose- and time-dependent manner^11^. In the current study, we explored the effect of P2Y_14_R on the regulation of pyroptosis. Consistent with previous studies, our data indicated that P2Y_14_R knockout effectively reduced the generations of inflammatory cytokines and inhibited the assembly of NLRP3 inflammasome. Therefore, the P2Y_14_R universally distributed in the immune system, might serve as a potential target for the different diseases therapy.

The air pouch model is a classic animal model commonly applied for studies on inflammatory diseases^35–37^. The air pouch cavity derived from consecutive air injection for 1 week, includes a layer of fibroblasts that resembles the human synovia structure. After dorsal air pouch formation, a recruitment of abundant macrophages occurred in response to the cavity injection of proinflammatory substances^38,39^. Subsequently, peritoneal macrophages were harvested for the following experiments by washing the cavity with sterile PBS solution. Given the fibroblasts disturbance in synovial tissue, we investigated the role of P2Y_14_R in macrophages collected by air pouch model and related data were shown in Supplementary Figure 5. Consistent with our synovial tissue data, P2Y_14_R knockout abolished the onset of MSU-induced pyroptosis stress in macrophages of air pouches, as insignificant difference in pyroptosis assay of P2Y_14_R knockout rats treated with or without MSU. It suggested that P2Y_14_R knockdown effectively inhibited the cell death process caused by MSU. Next, the cAMP was concerned about its mediator between P2Y_14_R and pyroptosis. An elevated cAMP production caused by the adenylate cyclase activator Forskolin, suppressed the Caspase-1-mediated pyroptosis in collected macrophages of wild-type rats. While, inhibition of AC-mediated cAMP synthesis by SQ22536 administration in P2Y_14_R knockout animals, disrupted the resistance to MSU stimulation with activated caspase-1 release and increased pyroptotic positivity, suggesting cAMP metabolism was involved in the regulation of P2Y_14_R in macrophages. And it was in macrophages that P2Y_14_R controlled the development of pyroptosis process via regulating adenylate cyclase-mediated cAMP synthesis.

**Fig. 7:**
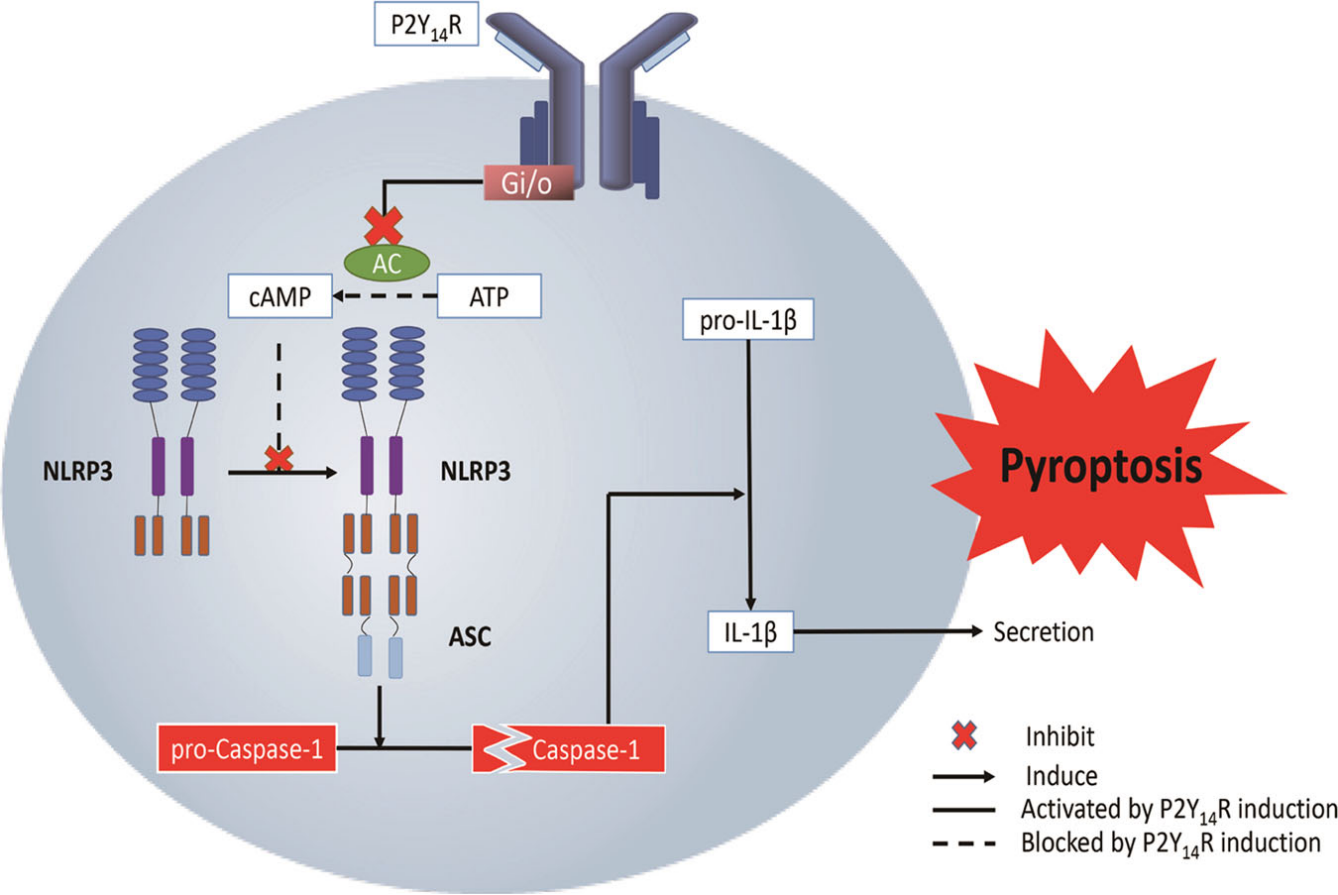
Proposed mechanism of P2Y_1__4_R involved in pathogenesis of acute gouty arthritis. P2Y_14_R regulates pyroptosis of macrophages through cAMP/NLRP3 signals.

In summary, the present study demonstrated the critical role of P2Y_14_R in the pathogenesis of acute gouty arthritis, which might be attributed to its effects on MSU-induced pyroptosis. Here, a unique role for intracellular cAMP was proposed in mediating crosstalk between P2Y_14_R activation and inflammatory cascade in acute gouty arthritis flares (Fig. 7). However, more studies would be necessary to clarity the mechanism behind P2Y_14_R-cAMP-NLRP3 inflammasome axis in future.

## Supplementary information

Supplementary Figure Legends

Supplementary Figure 1

Supplementary Figure 2

Supplementary Figure 3

Supplementary Figure 4

Supplementary Figure 5

Supplementary Tables
