# Supplementary material for: P2Y14 receptor has a critical role in acute gouty arthritis by regulating pyroptosis of macrophages
Source: Cell Death Dis. 2020 May 26;11(5):394. doi: 10.1038/s41419-020-2609-7 (PMC7250907; doi:10.1038/s41419-020-2609-7)
Supplement: Supplementary file 1 — Supplementary Figure Legends [file 41419_2020_2609_MOESM1_ESM.doc]

**Supplementary Figure and Table Legends**

**Supplementary Figure 1**. **P2Y_14_R knockout alleviated acute gouty arthritis in** **P2Y_14_R^-/-^ rats.** The acute gouty arthritis was induced by intra-articular injection with 100 μl MSU crystals (500 μg/ml). The expression of NLRP3 inflammasome activation and IL-1β in synovium was assayed by western blotting. The relative optical densities of NLRP3 (A), ASC (B), Caspase-1 (C) and IL-1β (D) were analyzed. Genotype identification of P2Y_14_R-KO rats (E). NC: Negative Control; PC: Positive Control; F2-01: Wild Type; F2-02: P2Y_14_R^+/-^; F2-03, F2-04: P2Y14R^-/-^. Compared with WT+vehicle group: ^#^*P*<0.05, ^##^*P*<0.01, ^###^*P*<0.001; Compared with WT+MSU group: ^*^*P*<0.05, ^**^*P*<0.01, ^***^*P*<0.001.

**Supplementary Figure 2**. **P2Y_14_R knockdown attenuated the pyroptosis.** P2Y_14_R siRNA was used to transfect THP-1 cells at a confluency of 70%-90% with Lipofectamine 2000. After transfection for 48 h, the THP-1 cells were stimulated by MSU (500 μg/ml) for 12 h. The expression of NRLP3 inflammasome activation was measured by western blotting. The relative optical densities of NLRP3 (A), ASC (B) and Caspase-1 (C) were analyzed (n=4). The efficiency of siRNA transfection into THP-1 cells (D). Compared with NC+vehicle group: ^#^*P*<0.05, ^##^*P*<0.01, ^###^*P*<0.001; Compared with NC+MSU group: ^*^*P*<0.05, ^**^*P*<0.01, ^***^*P*<0.001.

**Supplementary Figure 3**. **Increased cAMP suppressed pyroptosis stress.** As a potent adenylate cyclase (AC) activator, Forskolin is frequently used to increase the intracellular cAMP level. Intra-articular administration of Forskolin (10 mg/kg) was given to WT rats three times at 0, 24, 48 h prior to MSU induction. The relative expressions of NLRP3 (A), ASC (B), Caspase-1 (C) and IL-1β (D) were analyzed (n=4). Meantime, the effect of Forskolin was also investigated in THP-1 cells. 15 min pre-treatment of Forskolin (10 μM) was exposed to THP-1 cells followed by MSU model (500 µg/ml) for 12 h. The relative expressions of NLRP3 (E), ASC (F) and Caspase-1 (G) were exhibited (n=4). Compared with WT+vehicle group: ^#^*P*<0.05, ^##^*P*<0.01, ^###^*P*<0.001; Compared with WT+MSU group: ^*^*P*<0.05, ^**^*P*<0.01, ^***^*P*<0.001.

**Supplementary Figure 4**. **Inhibited cAMP level reversed the effect of** **P2Y_14_R deficiency.** SQ22536, an adenylate cyclase (AC) inhibitor, was used in our study to reduce cAMP levels in P2Y_14_R-KO rats. Intra-articular administration of SQ22536 (10 mg/kg) was given to P2Y_14_R-KO rats three times at 0, 24, 48 h prior to MSU model. The relative expressions of NLRP3 (A), ASC (B), Caspase-1 (C) and IL-1β (D) were analyzed (n=4). Compared with P2Y_14_R-KO+vehicle group: ^#^*P*<0.05, ^##^*P*<0.01, ^###^*P*<0.001. Compared with P2Y_14_R-KO+MSU group: ^*^*P*<0.05, ^**^*P*<0.01, ^***^*P*<0.001. Next, the effect of SQ22536 was also investigated in THP-1 cells transfected with siRNA. After transfection for 48 h, 15 min pre-treatment of SQ22536 (10 μM) was exposed to THP-1 cells followed by MSU model (500 µg/ml) for 12 h. The relative expressions of NLRP3 (E), ASC (F) and Caspase-1 (G) were shown (n=4). Compared with siP2Y_14_R+vehicle group: ^#^*P*<0.05, ^##^*P*<0.01, ^###^*P*<0.001; Compared with siP2Y_14_R+MSU group: ^*^*P*<0.05, ^**^*P*<0.01, ^***^*P*<0.001.

**Supplementary Figure 5**. **The regulation of P2Y_14_R occurred in macrophages.** The air pouch cavity was established by consecutive air injection for one week to collect peritoneal macrophages to investigate the effect of P2Y_14_R on pyroptosis. (A) Pyroptosis assay was conducted in the macrophages derived from WT rats treated with Forskolin (n=4). Compared with WT+MSU group: ^*^*P*<0.05, ^**^*P*<0.01, ^***^*P*<0.001. (B) The effect of SQ22536 on pyroptosis onset was also evaluated with active PI and Caspase-1 double staining in the macrophages collected from P2Y_14_R-KO rats (n=4). Compared with P2Y_14_R-KO+MSU group: ^*^*P*<0.05, ^**^*P*<0.01, ^***^*P*<0.001.

**Supplementary Table 1** Increments of rats ankle perimeter (cm). The perimeter of the ankle was measured at different intervals for 24 h. The data was presented as means ± SDs. Compared with WT+vehicle group, ^###^*P*<0.001; Compared with WT+MSU group, ^***^*P*<0.001 (n=6).

**Supplementary Table 2** Increments of rats ankle perimeter (cm). The perimeter of the ankle was measured at different intervals for 24 h. The data was presented as means ± SDs. Compared with WT+vehicle group, ^###^*P*<0.001; Compared with WT+MSU group, ^***^*P*<0.001 (n=6).

**Supplementary Table 3** Increments of rats ankle perimeter (cm). The perimeter of the ankle was measured at different intervals for 24 h. The data was presented as means ± SDs. Compared with P2Y_14_R-KO+MSU group, ^***^*P*<0.001 (n=6).
