# Supplementary figures and images for: P2Y14 receptor has a critical role in acute gouty arthritis by regulating pyroptosis of macrophages
Source: Cell Death Dis. 2020 May 26;11(5):394. doi: 10.1038/s41419-020-2609-7 (PMC7250907; doi:10.1038/s41419-020-2609-7)

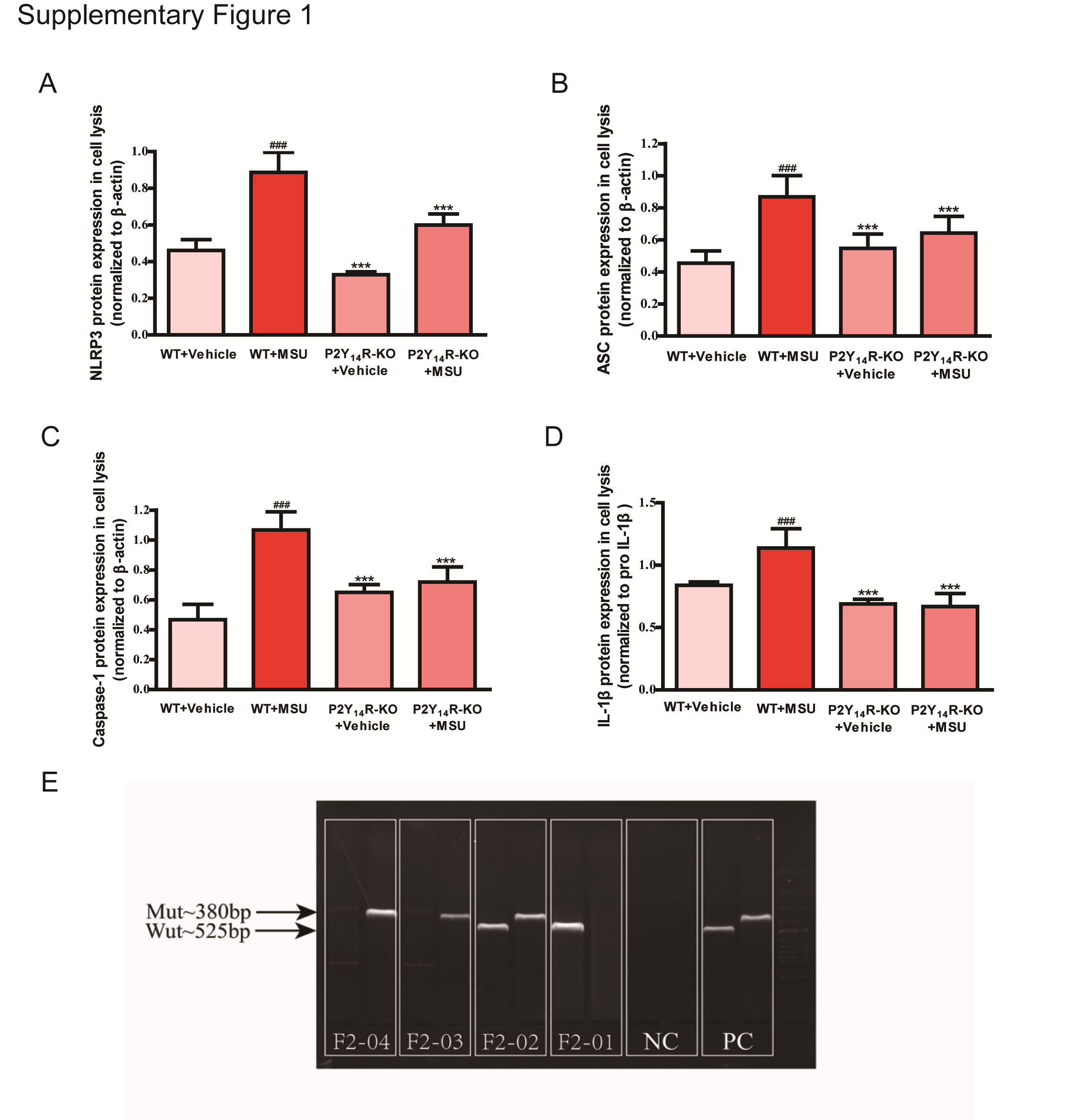

Supplement: Supplementary file 2 — Supplementary Figure 1 [file 41419_2020_2609_MOESM2_ESM.tif]

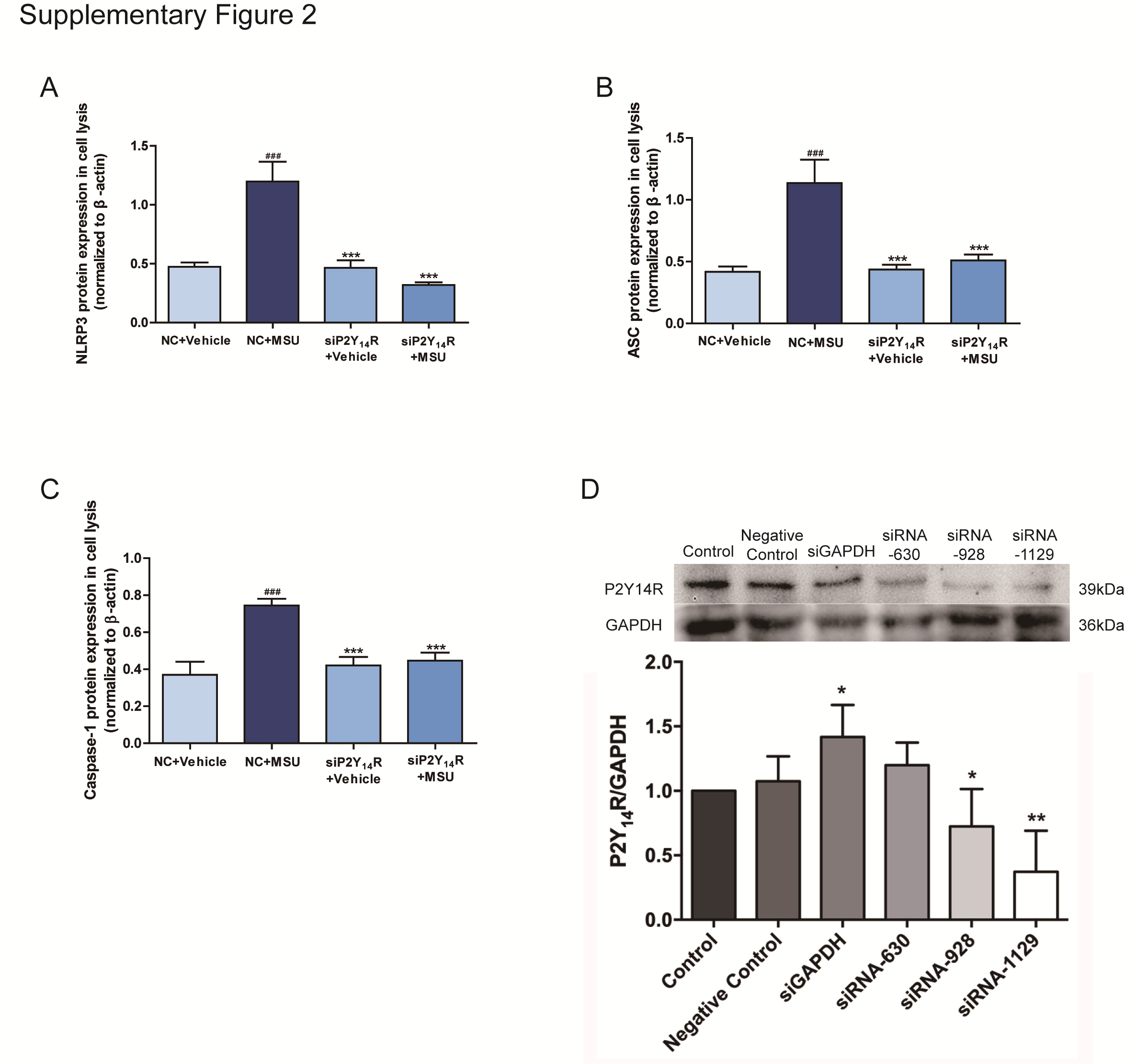

Supplement: Supplementary file 3 — Supplementary Figure 2 [file 41419_2020_2609_MOESM3_ESM.tif]

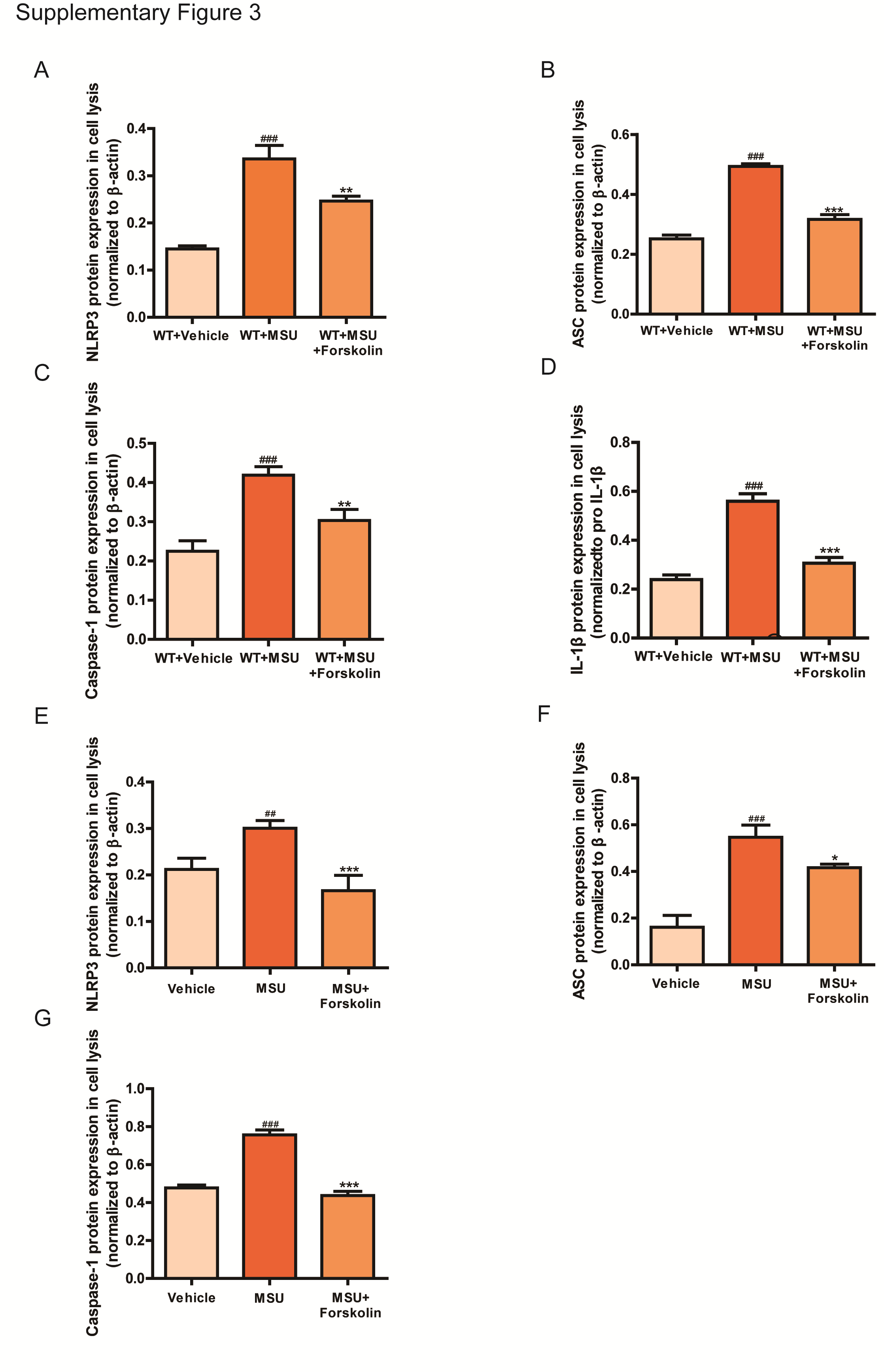

Supplement: Supplementary file 4 — Supplementary Figure 3 [file 41419_2020_2609_MOESM4_ESM.tif]

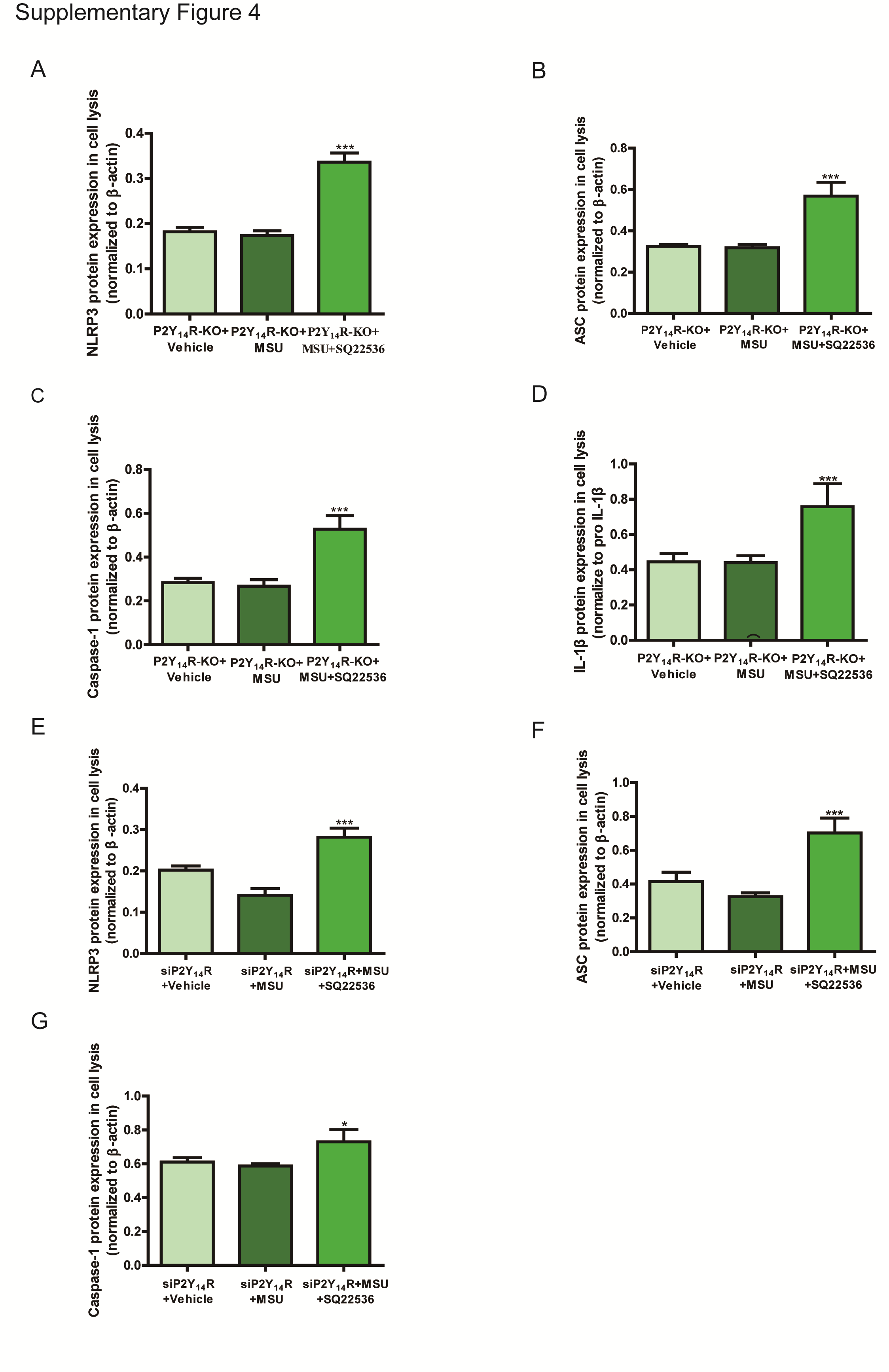

Supplement: Supplementary file 5 — Supplementary Figure 4 [file 41419_2020_2609_MOESM5_ESM.tif]

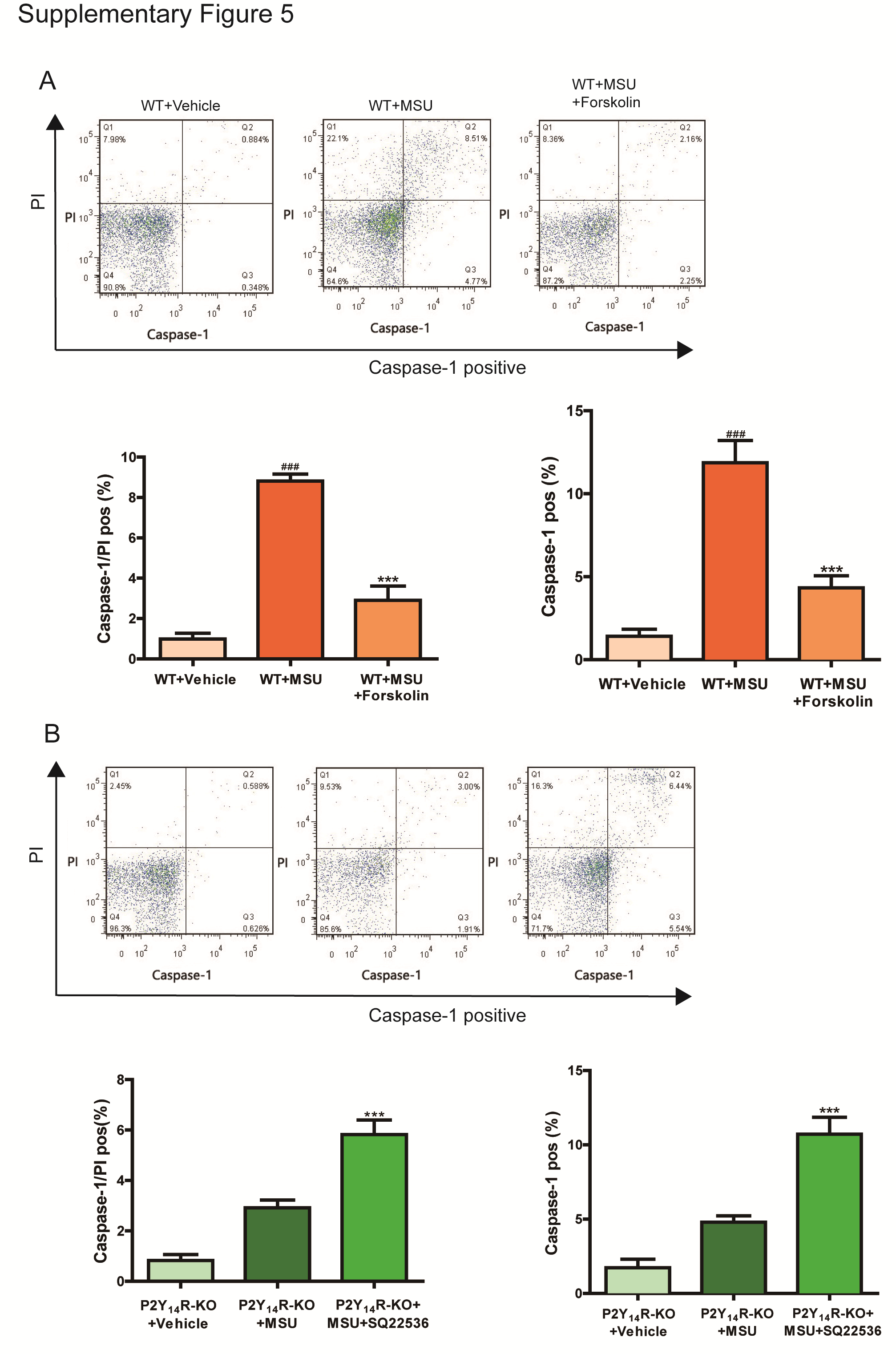

Supplement: Supplementary file 6 — Supplementary Figure 5 [file 41419_2020_2609_MOESM6_ESM.tif]
