# Supplementary material for: P2Y14 receptor has a critical role in acute gouty arthritis by regulating pyroptosis of macrophages
Source: Cell Death Dis. 2020 May 26;11(5):394. doi: 10.1038/s41419-020-2609-7 (PMC7250907; doi:10.1038/s41419-020-2609-7)
Supplement: Supplementary file 7 — Supplementary Tables [file 41419_2020_2609_MOESM7_ESM.doc]

**Supplementary Table 1 Increments of rats ankle perimeter (cm)**

|  | 2h | 4h | 8h | 12h | 24h |
| --- | --- | --- | --- | --- | --- |
| WT+Vehicle | 0.31±0.01 | 0.42±0.04 | 0.24±0.04 | 0.11±0.03 | 0.21±0.01 |
| WT+MSU | 0.73±0.02^###^ | 0.74±0.04^###^ | 0.97±0.07^###^ | 1.12±0.06^###^ | 0.97±0.03^###^ |
| P2Y_14_R-KO+Vehicle | 0.32±0.04^***^ | 0.44±0.01^***^ | 0.43±0.04^***^ | 0.39±0.03^***^ | 0.25±0.01^***^ |
| P2Y_14_R-KO+MSU | 0.37±0.04^***^ | 0.48±0.02^***^ | 0.60±0.02^***^ | 0.44±0.03^***^ | 0.35±0.03^***^ |

**Supplementary Table 2 Increments of rats ankle perimeter (cm)**

|  | 2h | 4h | 8h | 12h | 24h |
| --- | --- | --- | --- | --- | --- |
| WT+Vehicle | 0.32±0.05 | 0.48±0.06 | 0.52±0.02 | 0.31±0.04 | 0.28±0.06 |
| WT+MSU | 0.81±0.05^###^ | 1.12±0.04^###^ | 1.37±0.03^###^ | 1.55±0.07^###^ | 1.2±0.04^###^ |
| WT+MSU+Forskolin | 0.36±0.03^***^ | 0.46±0.04^***^ | 0.54±0.07^***^ | 0.27±0.07^***^ | 0.34±0.05^***^ |

**Supplementary Table 3 Increments of rats ankle perimeter (cm)**

|  | 2h | 4h | 8h | 12h | 24h |
| --- | --- | --- | --- | --- | --- |
| P2Y_14_R-KO+Vehicle | 0.20±0.01 | 0.29±0.01 | 0.28±0.04 | 0.15±0.02 | 0.23±0.01 |
| P2Y_14_R-KO+MSU | 0.30±0.09 | 0.39±0.05 | 0.30±0.05 | 0.10±0.06 | 0.16±0.02 |
| P2Y_14_R^-^KO+MSU+ SQ22536 | 0.46±0.01^***^ | 0.61±0.02^***^ | 0.72±0.02^***^ | 0.73±0.07^***^ | 0.89±0.07^***^ |
